# Annual Meeting of Alumni of Baltimore College of Dental Surgery

**Published:** 1879-05

**Authors:** 


The Annual Meeting of the Alumni of the Baltimore College of Dental Surgery, on the 6th of last month, was of the most pleasant character, and greatly enjoyed by all participating in it. There was more of heartiness in the reunion than is commonly seen; and the consolidation of the two colleges was the subject of many congratulatory remarks. The friends of both the old and the new institutions, cordially united, will throw the weight of their influence in the scale of the, comparatively speaking, venerable School, and the prediction is made that a new era of prosperity has begun. For the College in question, the editors of this Journal promise that the light of past experience will be allowed its full power of guidance for the future, and that the confidence reposed in the Faculty by the public in the past and present, will be, if faithful efforts will avail on our part, not unmerited. To our friends everywhere we say, send us carefully trained students, and select as your students, so far as you are able, men of culture. The future of dentistry lies largely in these last few lines. H.

